# Relationships of *H*. *pylori* infection and its related gastroduodenal morbidity with metabolic syndrome: a large cross-sectional study

**DOI:** 10.1038/s41598-018-22198-9

**Published:** 2018-03-06

**Authors:** Rotem Refaeli, Gabriel Chodick, Saeda Haj, Sophy Goren, Varda Shalev, Khitam Muhsen

**Affiliations:** 1 Department of Epidemiology and Preventive Medicine, School of Public Health, Sackler Faculty of Medicine, Tel Aviv University, Tel Aviv, Israel; 2 Medical division, Maccabi Health Services, Tel Aviv, Israel

## Abstract

The few published studies on the relationship between *Helicobacter pylori* infection and metabolic homeostasis were relatively small and yielded inconsistent results. We examined the prevalence of metabolic syndrome in relation to *H*. *pylori* infection and its symptoms in a large and unselected population. Coded data from the computerised database of a large health maintenance organisation in Israel were accessed for 147,936 individuals 25–95 years of age who performed the urea breath test during 2002–2012. The classification of metabolic syndrome followed a modified definition of the international diabetes federation. Prevalences of *H*. *pylori* infection and metabolic syndrome were 52.0% and 11.4% respectively. *H*. *pylori* infected patients had increased likelihood of metabolic syndrome: adjusted odds ratio (aOR) 1.15 (95% confidence intervals (CI) 1.10–1.19), as did patients with gastric ulcer: aOR 1.15 (95% CI 1.03–1.28) vs patients without these conditions. Duodenal ulcer was associated with metabolic syndrome only in persons aged 25–34 years: aOR 1.59 (95% CI 1.19-2.13), but not in older persons (P = 0.001 for heterogeneity). In conclusion, the likelihood of metabolic syndrome appeared significantly increased in relation to *H*. *pylori* infection and gastric and duodenal ulcers. These findings suggest that *H*. *pylori* long-term gastric inflammation might play a role in metabolic homeostasis.

## Introduction

Metabolic syndrome is a cluster of metabolic factors that increase the risk for atherosclerotic cardiovascular disease and type 2 diabetes mellitus^1^. Metabolic syndrome consists of atherogenic dyslipidaemia, elevated blood pressure; and elevated fasting blood glucose levels^2,3^, which lead to a prothrombotic and pro-inflammatory state^1^.

It has been postulated that some infectious agents might play a role in the pathogenesis of metabolic syndrome, as they can induce persistent inflammation. *Helicobacter pylori*, a gram-negative bacterium, is of particular interest, given its ability to induce long-standing gastric inflammation^4^. *H*. *pylori* colonizes the human gastric mucosa and causes persistent inflammation, usually without causing apparent disease, but in some individuals *H*. *pylori* causes peptic ulcer disease and even adenocarcinoma of the stomach^5,6^. The pathogenesis of peptic disease and gastric cancer usually involves strong and persistent inflammatory response that does not clear the infection (reviewed in^5^).

*H*. *pylori* infection has been linked to health conditions in areas outside the stomach, collectively known as extragastric diseases^7,8^. *H*. *pylori* infection was reported to be associated with increased prevalence of the metabolic syndrome^4,9–12^. However, conflicting findings exist regarding such association^12,13^, its magnitude^14^ and specificity^15^. Namely, it is unclear whether the observed association is specific to *H*. *pylori* infection or is the result of exposure to infectious agents in general, such as *Chlamydia pneumoniae*, herpes simplex virus 1 and cytomegalovirus^15^.

The aims of the current study were to examine the prevalence of metabolic syndrome in relation to *H*. *pylori* infection, and to peptic ulcer disease, as a proxy for gastric inflammation. If *H*. *pylori* infection and gastric inflammation are important in the development of metabolic syndrome, a stronger association is expected in symptomatic *H*. *pylori* patients.

## Results

Data for 147,936 persons (60.7% females) who performed the urea breath test (UBT) and met the inclusion criteria were analysed. The age at UBT ranged from 25–95 years, with a mean of 42.8 years (standard deviation (SD) 12.7). *H*. *pylori* tested positive by UBT in 76,965 (52.0%; [95% CI 51.8–52.3]) persons.

Overall, 104,626 persons (70.7%) had information on at least one parameter of metabolic syndrome (in addition to body mass index [BMI]); this percentage was significantly higher in the older age groups, residents of low socioeconomic status towns and those who were born abroad compared to persons who were born in Israel(Supplementary Table S1). Accordingly, we conducted the weighted analysis; yielding a 11.4% weighted prevalence of metabolic syndrome.

### Prevalence of metabolic syndrome according to socio-demographic factors

The prevalence of metabolic syndrome was higher in men than in women, it increased progressively with age (P for trend < 0.001), and decreased as residential economic rank increased (P for trend <0.001). Individuals who were born in Israel had lower prevalence of metabolic syndrome (9.7%) than those who were born in other countries (13.3–15.0%). Smokers and non-smokers had similar prevalence of metabolic syndrome (Table 1).

**Table 1:** Prevalence of metabolic syndrome according to sociodemographic factors.

	Total	Percent (weighted) with metabolic syndrome	P value
Sex			<0.001
Men	58,173	12.4%	
Women	89,763	10.8%	
Age, yearsa			<0.001
25–34	45,054	3.8%	
35–44	45,438	9.1%	
45–54	29,591	14.5%	
55–64	17,612	22.5%	
65–95	10,241	25.5%	
SES of town of residenceb			<0.001
1–5 (low)	60,282	13.7%	
6–7 (intermediate)	39,500	10.6%	
8–10 (high)	38,790	9.2%	
Missing	9364	8.9%	
Country of birthc			<0.001
Israel	96,180	9.7%	
Former Soviet Union	35,885	14.6%	
North Africa/Asia	5379	14.8%	
Europe/Americas	7009	15.0%	
Other/unknown	3483	13.3%	
Smoking			<0.001
Ever	20,913	11.9%	
Never	86,540	11.6%	
Unknown	40,483	10.7%	

^a^Degrees of freedom (df) = 4, P < 0.001 for trend; ^b^Df = 3, P < 0.001 for trend; ^c^Df = 4

SES: socioeconomic status.

### The prevalence of metabolic syndrome according to *H*. *pylori* infection, and gastric and duodenal ulcers

Overall, metabolic syndrome prevalence was slightly higher in *H*. *pylori* positive (by UBT) than negative persons (P < 0.001), but the difference was of greater magnitude in patients who had gastric and duodenal ulcers compared to those without a diagnosis code of these illnesses (Table 2). Adjusting for age resulted in strengthening the relationship of *H*. *pylori* with the metabolic syndrome, and attenuating the relationships of both gastric and duodenal ulcers with the metabolic syndrome.

**Table 2:** Unadjusted and age-adjusted associations of *H*. *pylori* infection and peptic disease with metabolic syndrome^a^.

	Total	Metabolic syndrome percentage (weighted)	OR (95% CI)	Age-adjusted OR_MH_ (95% CI)	P value^b^
*H*. *pylori* infection					<0.001
Negative	70,971	11.0%	Reference	Reference	
Positive	76,965	11.8%	1.07 (1.04–1.11)	1.18 (1.14–1.22)	
Gastric ulcer					<0.001
No	144,783	11.3%	Reference	Reference	
Yes	3153	16.2%	1.52 (1.38–1.67)	1.22 (1.10–1.34)	
Duodenal ulcer					<0.001
No	137,570	11.1%	Reference	Reference	
Yes	10,366	15.2%	1.44 (1.36–1.52)	1.11 (1.05–1.17)	

^a^CI: confidence intervals; OR: odds ratio; UBT: urea breath test.

^b^P value by Cochran’s Mantel-Haenszel test.

Stratification by age groups showed higher prevalences of metabolic syndrome in people with *H*. *pylori* infection, gastric ulcer or duodenal ulcer compared to people without these conditions in most age groups (Table 3).

**Table 3:** Age-specific weighted prevalence (%) of metabolic syndrome according to *H*. *pylori* infection, gastric ulcer and duodenal ulcer^a^.

	Age: 25–34 years			Age: 35–44 years			Age: 45–54 years			Age: 55–64 years			Age: 65–95 years		
	Total	MetS, %	P	Total	MetS, %	P	Total	MetS, %	P	Total	MetS, %	P	Total	MetS, %	P
*H*. *pylori* infection															
Negative	22,011	3.2%	<0.001	19,645	8.1%	<0.001	13,679	13.6%	<0.001	9592	21.8%	0.015	6044	25.0%	0.13
Positive	23,043	4.4%		25,793	9.9%		15,912	15.4%		8020	23.4%		4197	26.3%	
Gastric ulcer															
No	44,428	3.8%	0.6	44,702	9.1%	<0.001	28,861	14.5%	0.12	17,008	22.4%	0.01	9784	25.5%	0.4
Yes	626	3.5%		736	12.7%		730	16.5%		604	26.8%		457	27.0%	
Duodenal ulcer															
No	43,267	3.7%	<0.001	42,811	9.1%	0.018	27,114	14.3%	0.002	15,591	22.5%	0.8	8787	25.7%	0.3
Yes	1787	6.5%		2627	10.3%		2477	16.7%		2021	22.3%		1454	24.6%	

^a^MetS: metabolic syndrome.

Pooled and age-stratified multivariable analyses were fitted. The pooled analyses showed that *H*. *pylori* infected persons had significantly increased likelihood of metabolic syndrome compared to uninfected patients; adjusted odds ratio (aOR) 1.15 [95% CI 1.10–1.19]). The strength of association between gastric ulcer and metabolic syndrome was weakened following adjustment for age, country of birth and residential economic rank (Table 4). Similarly, the relationship between duodenal ulcer and metabolic syndrome was of weaker magnitude after adjusting for the abovementioned sociodemographic factors. Positive adjusted associations (P < 0.05) remained in the age groups 25–34, 35–44 and 45–54 years, among whom *H*. *pylori* infected persons had 1.20 and 1.18 and 1.12-fold increased likelihood, respectively, to have metabolic syndrome. Persons aged 25–34 who had duodenal ulcer had 1.56 higher odds for metabolic syndrome than persons without duodenal ulcer, P = 0.001 for heterogeneity by age (Table 4). The adjusted association between gastric ulcer and metabolic syndrome remained significant in the pooled analysis (aOR 1.15 [95% CI 1.00-1.28]) of all age groups; the associations was stronger in the age group 35–44 (aOR 1.38 [95% CI 1.08–1.77]) and 55–64 (aOR 1.24 [95% 1.00–1.51]), respectively (Table 4), but the heterogeneity test was not significant.

**Table 4:** Adjusted odds ratios and 95% confidence intervals of the associations of *H*. *pylori* infection, and gastric and duodenal ulcers, with metabolic syndrome, by age groups^a^.

	Overall	Age: 25–34	Age: 35–44	Age: 45–54	Age: 55–64	Age: 65–95	P for heterogeneity by age^d^
	OR (95% CI)	OR (95% CI)	OR (95% CI)	OR (95% CI)	OR (95% CI)	OR (95% CI)	
*H*. *pylori* positive^b^	1.15 (1.10–1.19)	1.20 (1.03–1.41)	1.18 (1.09–1.27)	1.12 (1.04–1.20)	1.05 (0.98–1.14)	1.04 (0.95–1.14)	0.11
Gastric ulcer^c^	1.15 (1.03–1.28)	0.81 (0.46–1.45)	1.38 (1.08–1.77)	1.09 (0.88–1.34)	1.24 (1.03–1.51)	1.05 (0.85–1.31)	0.2
Duodenal ulcer^c^	1.04 (0.98–1.11)	1.59 (1.19–2.13)	1.12 (0.97–1.29)	1.08 (0.96–1.22)	0.95 (0.84–1.07)	0.87 (0.76–0.99)	0.001

CI: confidence intervals; OR: Odds ratio.

^a^Each model adjusted for age (in years as a continuous variable), country of birth and socioeconomic status of town of residence; ^b^*H*. *pylori* positive vs negatives (by urea breath test); ^c^yes vs no.

^d^Degrees of freedom = 4 for chi square heterogeneity test.

## Discussion

We examined the prevalence of metabolic syndrome in relation to *H*. *pylori* infection and peptic ulcers. Overall, *H*. *pylori* infected persons had higher prevalence of metabolic syndrome than uninfected ones. Similarly, a positive association was found between gastric ulcer and the metabolic syndrome. The strength of these associations was modest. The associations of duodenal ulcer was significantly modified by age, showing significantly 1.59-fold increased likelihood to have metabolic syndrome, in patients with versus without this condition, only in the age group 25–34 years. Collectively these findings suggest complex relationships between infection with *H*. *pylori* infection and the metabolic syndrome, as well as of gastric and duodenal ulcers with the metabolic syndrome. Sociodemographic confounders appear to explain such associations only partially, thus suggesting that *H*. *pylori* might have an independent and specific role in the metabolic syndrome.

A limited number of investigations have assessed the prevalence of the metabolic syndrome according to infection with *H*. *pylori*^11–18^. Mostly, they were conducted in East Asia^11,12,14,16–18^ and two studies were undertaken in Middle Eastern countries (Lebanon and Iran)^13,15^. Except for Naja *et al*.^13^, all the studies demonstrated significantly higher prevalence of the metabolic syndrome in persons infected with *H*. *pylori* compared to uninfected ones^11,12,14–18^. The sample size in these studies ranged from 308 to 7394 persons. They employed various detection techniques of *H*. *pylori* and various classification criteria for the metabolic syndrome. Most of these studies reported results from adjusted models. A recent meta-analysis combining results from these studies^19^ yielded a pooled OR of 1.34 (95% CI 1.17–1.53), with significant heterogeneity across the studies^19^. Our study provides the largest sample size to date, in which we used a uniform classification of both the exposure and outcome variables, as well as adjusted for possible confounders. The magnitude of the association in our study was modest with an aOR of 1.15 (95% CI 1.1–1.19). A similar association was found in relation to gastric ulcer, while duodenal ulcer showed a stronger association in the age group 25–34 years. These illnesses are caused by *H*. *pylori* through persistent gastric inflammation induced by the bacterium^5^. Our observations are in agreement with findings reported by Chen *et al*.^17^, who demonstrated significantly increased likelihood of the metabolic syndrome in persons positive versus negative for *H*. *pylori* IgG serum antibody in young adults, although the magnitude of association in our study is much smaller. Shin *et al*. have shown^14^ a stronger association between *H*. *pylori* infection with metabolic syndrome, if the infection status was determined based on histology, but they found no significant association when using serological assay. Collectively, these and our findings suggest that *H*. *pylori* infected persons had higher prevalence of metabolic syndrome than uninfected ones. The association between gastroduodenal disease and the metabolic syndrome might suggest that more severe gastric inflammation might be involved in metabolic imbalance. Indeed, it was shown that *H*. *pylori* infection, and eradication of the infection, can alter ghrelin and leptin levels^20,21^ (reviewed by Haj *et al*.^4^), two hormones expressed in the stomach and involved in metabolic homoeostasis^22,23^. Additionally, it is possible that the long-term gastric inflammation might enhance the pro-inflammatory state related to metabolic syndrome.

Given the cross-sectional study design, we cannot determine whether observed associations are causal; nonetheless, they are of public health importance, as *H*. *pylori* infection and peptic diseases might serve as markers to identify patients with increased likelihood for metabolic syndrome. The interaction between age and peptic ulcer might be due to poorer survival of old symptomatic *H*. *pylori* infected patients, compared to survivors who were included in the cohort.

Limitations of our study should be acknowledged. Electronic health and demographic data from a health maintenance organisation (HMO) database were utilized. These data accumulate as part of patients’ care; specifically, in our study, in regard to patients who were referred to UBT by their physicians. Thus, our sample represents patients with gastrointestinal symptoms. Data collection on variables such as BMI and smoking might be different in the various clinics across the country. Other than smoking, information was missing in low percentages for such confounders as country of birth, socioeconomic status and BMI. Importantly, the study database lacks information on waist circumference; a surrogate marker of central obesity, therefore, we used obesity (BMI ≥30 kg/m^2^) as a proxy for central obesity. The cutoffs of waist circumference of 102 cm and 88 cm for men and women, respectively, are associated with increased risk of cardiovascular diseases, and they were driven by correlation with BMI >30 (^24^, reviewed in^25^). BMI and waist circumference are strongly correlated with correlations coefficients ranging from 0.80 to 0.95^26^. Therefore, if misclassification of metabolic syndrome exists, because of using BMI ≥30 kg/m^2^ as a proxy for central obesity, it is expected to be non-differential, thus suggesting that our findings are conservative.

In addition, non-differential misclassification of gastric ulcer and duodenal ulcer might have occurred since the presence of these conditions was determined based on diagnostic codes in medical records. Diagnosis code of peptic ulcers disease showed a high positive predictive value of >90%^27,28^. Such misclassification might have led the measure of association towards the null hypothesis.

Strengths of our study include using UBT results to determine the presence of *H*. *pylori* infection. UBT is highly sensitive and specific for *H*. *pylori* identification compared to gold standard tests^29^. The study sample size was very large, which allowed identification of a relatively modest association between infection with *H*. *pylori* and metabolic syndrome prevalence, with effect modification by age. In addition, the definition of diabetes mellitus^30^ and hypertension^31^ relied on the same criteria throughout the study period. These definitions combine physicians’ diagnoses, results of blood biochemical analyses (e.g., fasting blood glucose and glycated haemoglobin in diabetes), buying medications to treat these conditions (antidiabetic and blood lowering agents), and several blood pressure measurements. Moreover, a standard definition was employed for the metabolic syndrome. Lastly, all laboratory tests were performed in one laboratory during the study period.

In conclusion, persons infected with *H*. *pylori* and those with peptic ulcer disease have significantly higher likelihood of the metabolic syndrome compared to persons without these conditions. These findings suggest that *H*. *pylori* long-term gastric inflammation might play a role in metabolic homeostasis.

## Methods

### Study design and population

A prevalence study was undertaken employing health and demographic information obtained from the computerised database of Maccabi Health Services (MHS) HMO. MHS is ranked as the second largest HMO in Israel, insuring about two million people, i.e., about one-quarter of the population. The study cohort has been described in detail^32^. Briefly, we accessed deidentified information of persons aged 25 years or older who underwent UBT during 2002–2012. Persons with bariatric surgery and cancer diagnosis (within two years of the UBT) and documented purchase of anti-*H*. *pylori* therapy or proton pump inhibitors within one month of the UBT were excluded from the study.

### Definitions of the study variables

Sociodemographic data were collected on patients’ age (grouped as 25–34, 35–44, 45–54, 65–95 years), sex, town of residence and country of birth (grouped as Israel, North Africa/Asia, Former Soviet Unions, Europe/Americas and other).

The definition of socioeconomic status relied on residential economic rank; i.e. of town of residence^33^. This aggregative index reflects certain features of a specific geographical area, including income, housing, cars (i.e. motorization level), education and employment^33^. Persons living in towns with residential economic ranks of 1–5 were categorised as living in low socioeconomic areas, and those living in towns with 6–7 and 8–10 socioeconomic ranks were categorised as living in intermediate and high socioeconomic areas, respectively.

Persons with a UBT result >3.5 per thousand were classified as infected with *H*. *pylori*, and those having lower UBT results were classified as negatives or uninfected. The international Classification of Disease codes-9^th^ revision with clinical modifications (ICD-9) was employed to determine whether patients had gastric ulcer or duodenal ulcers.

Data were collected on smoking (categorised as never smoked, ever smokers, and smoking status unknown) and on BMI, which is calculated as weight in kilograms (kg)/height^2^ in meters (m)).

Laboratory results were obtained on triglycerides, high-density lipoproteins (HDL), and fasting plasma glucose levels. Data on purchasing statins and anti-diabetic medications was also retrieved. A modified classification of the International Diabetes Federation^1,34^ was employed to identify persons with metabolic syndrome. Obesity defined as BMI ≥30 kg/m^2^ was used as a proxy for central obesity given the lack of information on waist circumference. Obesity in addition to two or more of the following criteria defined metabolic syndrome: raised triglycerides (>150 mg/dL or statins use); low HDL levels (<40 mg/dL in men and <50 mg/dL in women), hypertension (defined based on MHS registry^31^); or raised plasma glucose levels (≥100 mg/dL or a diagnosis of diabetes based on MHS registry^30^).

### Statistical analysis

Differences in sociodemographic factors between patients with and without information on at least one parameter of metabolic syndrome (beyond BMI) were examined using chi square test. Patients who had information on metabolic syndrome parameters differed significantly in sociodemographic variables compared to patients lacking such information (Supplementary Table 1), therefore we used inverse probability weighting method^35^, where the weights were assigned as the inverse probability obtained from multiple logistic regression model predicting having information on metabolic syndrome parameters.

Differences in the prevalence of the metabolic syndrome according to sociodemographic variables, *H*. *pylori* infection and its related gastroduodenal diseases were examined by chi square test. Age-adjusted associations between the infection and metabolic syndrome were examined using Cochran’s Mantel-Haenszel test.

Using Generalized Estimating Equations we examined adjusted relationships of *H*. *pylori* infection (determined as UBT result >3.5), gastric ulcer and duodenal ulcer with metabolic syndrome, while adjusting for sociodemographic factors. Adjusted ORs (and 95% confidence intervals [CIs]) were obtained from these models. Pooled and aged stratified analyses were performed. Heterogeneity across age groups was assessed using the chi square test for heterogeneity. The significance level was determined as two-sided P < 0.05. Analysis of data was performed using IBM-SPSS version 23 (IBM, Armonk, New York, USA) and Winpepi^36^.

### Ethical consideration

Ethical approval was obtained from the institutional review board (IRB) (Helsinki committee) of Assuta Medical Centre and from the ethics committee of Tel Aviv University. An exempt from informed consent was granted due to the retrospective study design and the use of de-identified data (anonymized) from medical files. The study was conducted in accordance with the ethical guidelines of the Declaration of Helsinki.

### Data availability statement

The data used in our study accumulated as part of patients’ care. These data were not collected for research purposes. The datasets cannot be made publicly available, and access to individual level data is not possible, since lawful and ethical limitations exist for secondary usage of these data in research.

## Electronic supplementary material

Supplementary table 1
