# Supplementary material for: Relationships of H. pylori infection and its related gastroduodenal morbidity with metabolic syndrome: a large cross-sectional study
Source: Sci Rep. 2018 Mar 6;8:4088. doi: 10.1038/s41598-018-22198-9 (PMC5840265; doi:10.1038/s41598-018-22198-9)
Supplement: Supplementary file 1 — Supplementary table 1 [file 41598_2018_22198_MOESM1_ESM.pdf]

Relationships of *H. pylori* infection and its related gastroduodenal morbidity with metabolic syndrome: a large cross-sectional study

Rotem Refaeli [a], Gabriel Chodick [a,b], Saeda Haj [a], Sophy Goren [a], Varda Shalev [a,b], Khitam Muhsen [a *]

[a] Department of Epidemiology and Preventive Medicine, School of Public Health, Sackler Faculty of Medicine, Tel Aviv University, Tel Aviv, Israel.

[b] Medical division, Maccabi Health Services, Tel Aviv, Israel.

Supplementary table 1: Having information on one parameter of metabolic syndrome (in addition to body mass index) by sociodemographic variables

	Total	Tested, n (%)	P value
**Overall**	146,936	104,626 (70.7%)	
**Sex**			
Men	58,173	41,079 (70.6%)	0.4
Women	89,763	63,547 (70.8%)	
**Age, years**			
25-34	45,054	19,306 (42.9%)	<0.001
35-44	45,438	33,182 (73.0%)	
45-54	29,591	26,093 (88.2%)	
55-64	17,612	16,008 (90.9%)	
65-95	10,241	10,037 (98.0%)	
**SES of town of residence** [a]			
1-5 (low)	60,282	43,980 (73.0%)	<0.001
6-7 (intermediate)	39,500	28,392 (69.7%)	
8-10 (high)	38,790	26,168 (67.5%)	
Missing	9364	6086 (65.0%)	
**Country of birth**			<0.001
Israel	96,180	58,249 (60.6%)	
Former Soviet Union	35,885	32,211 (89.8%)	
North Africa/Asia	5379	4970 (92.4%)	
Europe/Americas	7009	6168 (88.0%)	
Other/unknown	3483	3028 (86.9%)	

[a] SES: socioeconomic status
